# Paradoxical effects of statins on endothelial and cancer cells: the impact of concentrations

**DOI:** 10.1186/s12935-023-02890-1

**Published:** 2023-03-10

**Authors:** Yasin Ahmadi, Javad Khalili Fard, Dlzar Ghafoor, Ali H. Eid, Amirhossein Sahebkar

**Affiliations:** 1 College of Science, Department of Medical Laboratory Sciences, Komar University of Science and Technology, 46001, Sulaymania, Iraq; 2 Department of Pharmacology and Toxicology, Faculty of Pharmacy, Tabriz University of Medical Sciences, Tabriz, Iran; 3 Department of Basic Medical Sciences, College of Medicine, QU Health, Qatar University, Doha, Qatar; 4 Biotechnology Research Center, Pharmaceutical Technology Institute, Mashhad University of Medical Sciences, Mashhad, Iran; 5 Applied Biomedical Research Center, Mashhad University of Medical Sciences, Mashhad, Iran; 6 Department of Biotechnology, School of Pharmacy, Mashhad University of Medical Sciences, Mashhad, Iran

**Keywords:** Statins, Akt, Apoptosis, Cancer, Cytostatic, Endothelial cells, Senescence

## Abstract

In addition to their lipid-lowering functions, statins elicit additional pleiotropic effects on apoptosis, angiogenesis, inflammation, senescence, and oxidative stress. Many of these effects have been reported in cancerous and noncancerous cells like endothelial cells (ECs), endothelial progenitor cells (EPCs) and human umbilical vein cells (HUVCs). Not surprisingly, statins' effects appear to vary largely depending on the cell context, especially as pertains to modulation of cell cycle, senescence, and apoptotic processes. Perhaps the most critical reason for this discordance is the bias in selecting the applied doses in various cells. While lower (nanomolar) concentrations of statins impose anti-senescence, and antiapoptotic effects, higher concentrations (micromolar) appear to precipitate opposite effects. Indeed, most studies performed in cancer cells utilized high concentrations, where statin-induced cytotoxic and cytostatic effects were noted. Some studies report that even at low concentrations, statins induce senescence or cytostatic impacts but not cytotoxic effects. However, the literature appears to be relatively consistent that in cancer cells, statins, in both low or higher concentrations, induce apoptosis or cell cycle arrest, anti-proliferative effects, and cause senescence. However, statins’ effects on ECs depend on the concentrations; at micromolar concentrations statins cause cell senescence and apoptosis, while at nonomolar concentrations statins act reversely.

## Introduction

Statins are the most widely used medications in the management and treatment of hypercholesterolemia. Based on their polarity, statins are categorized in two groups, lipophilic and hydrophilic statins. Lipophilic statins readily cross the cell membrane, whereas hydrophilic ones employ carrier proteins to gain intracellular access. Lipophilic statins include atorvastatin, lovastatin, simvastatin, pitavastatin, and fluvastatin, while pravastatin and rosuvastatin are hydrophilic ones [1–4]. Considering their simple diffusion through membrane, the pleiotropic effects of lipophilic statins in extrahepatic tissues are extensively studied [5–18]. In particular, simvastatin has been widely used in cancer studies [19]. Owing to their inability to readily cross cell membranes, hydrophilic statins do not have pleiotropic effects in extrahepatic cells or their effects are not significant (see Tables 1 and 2) [20–22]. This could explain why they are less investigated in cancer studies as compared to lipophilic ones.

**Table 1:** The effects of statins on apoptosis and senescence in different vascular cardiovascular cell lines

Statin	Dose/concentration	Cell type	Mechanism of effect	Effect on apoptosis
Fluvastatin Pravastatin	3 µM 10–20 µM	Cardiac myocytes of Sprague–Dawley rats	Inhibition of the RhoA localization in the membrane; pravastatin did not have effect [101]	Increased
Simvastatin Fluvastatin Pravastatin	Animal Rat fluvastatin 50 mg/kg per day	L6 fibroblast cells and Rats in vivo	Inhibition of the RhoA localization in the membrane; pravastatin did not have effect [102]	Increased (cell line) Myotoxicity (Rat)
Simvastatin	71.6 µM, 143.3 µM	L6 myoblasts	Induction of tyrosine phosphorylation, 60 µM of pravastatin did not have effect on apoptosis [103]	Increased **(**71.6 µM**),** 143 µM cause necrosis
Simvastatin	71.6 µM	L6 myoblasts	Inhibition of the Ras isoprenylation and its downstream pathway Raf1/MEK and PI3-k [104]	Increased
Simvastatin	Oral administration (50 mg/kg/day) for 2 weeks,	Animal rabbit cardiac fibers	Raised serum CK and myopathy was induced by lesions of the muscle surface membrane [105]	Increased (myopathy)
Simvastatin, Simvastatin-acid form, and pravastatin	Simvastatin 47.8 µM, 60 µM, 71.6 µM simvastatin-acid 401 μM pravastatin μg/ml	L6 rat myoblasts	The mechanism of cell damage may relate to the [Ca2 +]i releasing and lipophilicity: Pravastatin caused little or no change in [Ca2 +]i and cell damage while simvastatin induced Apoptosis [106]	Increased by simvastatin Lipophilic statin but not pravastatin hydrophilic statin
Fluvastatin Pitavastatin Pravastatin	1–10 µM	Synoviocytes	Blocking geranylgeranylation of RhoA and subsequently activation of caspase 3; pravastatin had no effect [107]	Increased
Atorvastatin Simvastatin	100 μM	Vascular smooth muscle cells from Rat thoracic aorta	Blocking the prenylation of RhoA and downregulating the expression of Bcl-2 [78]	Increased
Simvastatin Fluvastatin Pravastatin	0.5–5 μM	Primary human adult cardiac myocytes	Downregulating both mRNA/protein of Mcl-1 (an inhibitor of apoptosis); pravastatin had no effect [108]	Increased
Atorvastatin Mevast	0.01 to 0.1 µM	HUVECs	Activate the endothelial Ras and promote Akt and mediate activation of eNOS [69]	Increased
Atorvastatin Mevastatin	> 0.1 µM	EPCs and Mononuclear cells	Halt angiogenesis and induce endothelial cell apoptosis [69]	Not seen even at high doses
Atorvastatin Mevastatin	0.1, 0.05, and 0.01 µM	EPCs and Mononuclear cells	Inhibit senescence [81] Regulation of cell cycle regulatory genes [81]	decreased
Atorvastatin, Pravastatin Pitavastatin	nanomolar concentrations	HUVEC	Inhibit senescence via activation of Akt and then upregulation of eNOS, SIRT1, and catalase [83]	Decreased
Simvastatin pravastatin	(0.1 µM)	In vivo: rabbit In vitro: HUVEC, COS-7 cells	Activation of Akt/eNOS and consequently induction of angiogenesis [109]	
Lovastatin	2, 10, and 50 μM	In vitro Mononuclear cells (MNCs) CD34 + isolated from human umbilical cord	Lovastatin reverses the survival and function of EPCs by regulating the Akt/eNOS signaling pathway and the gene transcription of eNOS. Coincubation of 50 µM simvastatin with Triciribine induced apoptosis [110]	Inhibition of the apoptosis induced by oxLDL
Atorvastatin Rosuvastatin	0.01–1 μM	EPCs isolated from peripheral blood	Atorvastatin suppressed homocysteine-induced ROS accumulation and EPCs apoptosis. It also antagonized Hcy-induced activation of NADPH oxidase and overexpression of Nox4 mRNA and p-p38MAPK protein. Nox4 siRNA transfected EPCs showed a similar result [111, 112]	Decreased
Pravastatin	0.002, 0.02, 0.2, 2 μM	Endothelial colony-forming cells (ECFCs)	Akt- and eNOS-phosphorylation were augmented. Further, expression levels of HO-1, VEGF-A, and PlGF were increased, whereas expression levels of sFlt-1 and Eng were decreased [113]	Proliferation, migration, and tube formation of ECFCs were enhanced by pravastatin
Atorvastatin Mevastatin	0.1 μM 0.1 μM	EPCs	Upregulation of the telomere repeat-binding factor TRF2 [114]	Statins enhance the migratory capacity of EPCs
Atorvastatin Mevastatin	0.1 μM	EPCs	Atorvastatin or mevastatin dose-dependently inhibited the onset of EPC senescence in culture. Moreover, atorvastatin increased the proliferation of EPCs. Atorvastatin modulated the expression of cell cyclins while downregulating the cell cycle inhibitor, p27Kip1. The effects of statins on the senescence were independent of NO, ROS, telomerase, and Rho kinase but dependent on GGPP [115]	statins inhibited senescence of EPCs

**Table 2:** The effects of statins on the apoptosis and senescence in different cancer cell lines

Statin	IC50 values	Cell line	Mechanism of effect	Effect on apoptosis
Fluvastatin	10 μM	MCF10A	The anticancer effect of statins is independent of prenylation of RAS family proteins and is associated with a cancer cell EMT phenotype [126] Inhibition of the RAS prenylation was uncoupled from fluvastatin-induced-apoptosis [126]	Induction of apoptosis
Simvastatin Atorvastatin	Atorvastatin 0.3–49.1 µM, Simvastatin 0.2- 40.8 µM	Triple-negative breast cancer (TNBC)	MVA rescued the effects of these statins [127]	Induction of apoptosis
Simvastatin	12 and 8 μM	12 and 8 μM in PC3 and LNCaP cell lines respectively	Induces subG1/G1 arrest [128]	Induction of apoptosis
	4.06 µM	breast cancer cell lines BoM-1833 (BoM) derived from MDA-231, MCF7/BoM, and T47D	Growth inhibition [129]	Induction of apoptosis
Simvastatin	0.481 µM	Adrenal carcinoma SW13 vimentin-positive (SW13-vim^+^)	Simvastatin targeting of vimentin may promote apoptotic cell death [130]	Induction of apoptosis
Simvastatin	60 µM	T47D breast cancer cell	Decreased the cyclin D1 expression and cell growth [131]	Induction of apoptosis
Atorvastatin	1.16 μM to 4.3 μM	MDA-MB-231 cells	Atorvastatin sensitivity correlates with decreased cholesterol levels in atorvastatin-treated cell [132]	
Simvastatin, fluvastatin		PC9 and PC9 GR4 (simvastatin:4 µM, fluvastatin: 2 µM) H460, H358, and PC9 BrM3 (simvastatin:12 µM, fluvastatin: 4 µM)	Decreases metastatic lung cancer cell survival *in vitro* synergistically with ABL tyrosine kinases inhibitor. Isoprenoid and mevalonate rescued the effects of statin [133]	Induction of apoptosis
Fluvastatin	5.3 µM	human A549 lung adenocarcinoma cells	PI3K inhibition [134]	Induction of apoptosis
Lovastatin		Anaplastic thyroid cancer cells	Blocking the membrane localization of RhoA and Rac. Mevalonate, GGPP rescued the effects of these statin [135]	Induction of apoptosis
Lipophilic statins		Osteosarcoma cells	Blocking RhoA-p42/p44 MAPKs-Bcl-2 survival pathway. RhoA agonist rescued the effects of statins [122]	Induction of apoptosis
Lovastatin	0.3 µM	Human prostate cancer cells	Induce senescence and cause G1 cell cycle arrest. GGPP/mevalonate, but not FPP were able to rescue the effects of statin [124]	Induction of apoptosis
Pitavastatin	10 μM	Breast cancer and melanoma tumors	Enhance effects of radiation on cellular senescence of radiation. 5 mM mevalonate rescued the effects of Pitavastatin [30]	Induction of apoptosis
Simvastatin	0.1 µM	Primary prostatic Normal epithelial cell lines RWPE-1 and PWR-1E	Exert cytostatic and senescent effects and partially induced apoptosis [125]	Induction of apoptosis
Simvastatin	10 μM	Primary prostatic normal epithelial cell lines RWPE-1 and PWR-1E cancer cells	In contrast, simvastatin had a cytotoxic effect both on normal and cancer cells. Combination of LDL-C and mevalonate rescued the effects of statin [125]	Induction of apoptosis
Lovastatin	0.3 µM	Prostate cancer cells: PC-3, DU-145, LNCaP	Senescence and G1 cell cycle arrest. GGPP, mevalonate, constitutively active RhoA (caRhoA) rescued the effects of lovastatin [124]	Induction of apoptosis

In addition to inhibiting cholesterol synthesis, statins can also reduce non-sterol products of the mevalonate pathway, such as isoprenoids [23–30]. Farnesyl pyrophosphate (FPP) and geranylgeranyl pyrophosphate (GGPP), are the major isoprenoids involved in prenylation of proteins [31]. Several proteins ranging from heterotrimeric G protein subunits to nuclear lamins have been found to undergo prenylation. However, the Ras superfamily of small GTPases is the most widely-known group of these proteins [32]. In this paper, we discuss some of the most important signaling pathways that are modulated by statins.

The Ras group of proteins plays a significant role in cell growth, proliferation, and survival [33–36]. Two major Ras-driven signaling cascades are the MAPK (Raf/MEK/ERK) and PI3K/Akt/mTOR pathways, both of which regulate cellular proliferation and differentiation [34, 37–40]. The MAPK pathway promotes proliferation by activating several transcription factors and kinases including AP-1, Myc, Jun, Fos, p90RSK1, Elk, Ets, and MNK [34, 41–44]. Moreover, it regulates survival and apoptosis by modulating the activity of several proteins like including JNK, SAPK, 14-3-3, and NF-Kβ [45, 46].

**Fig. 1:**
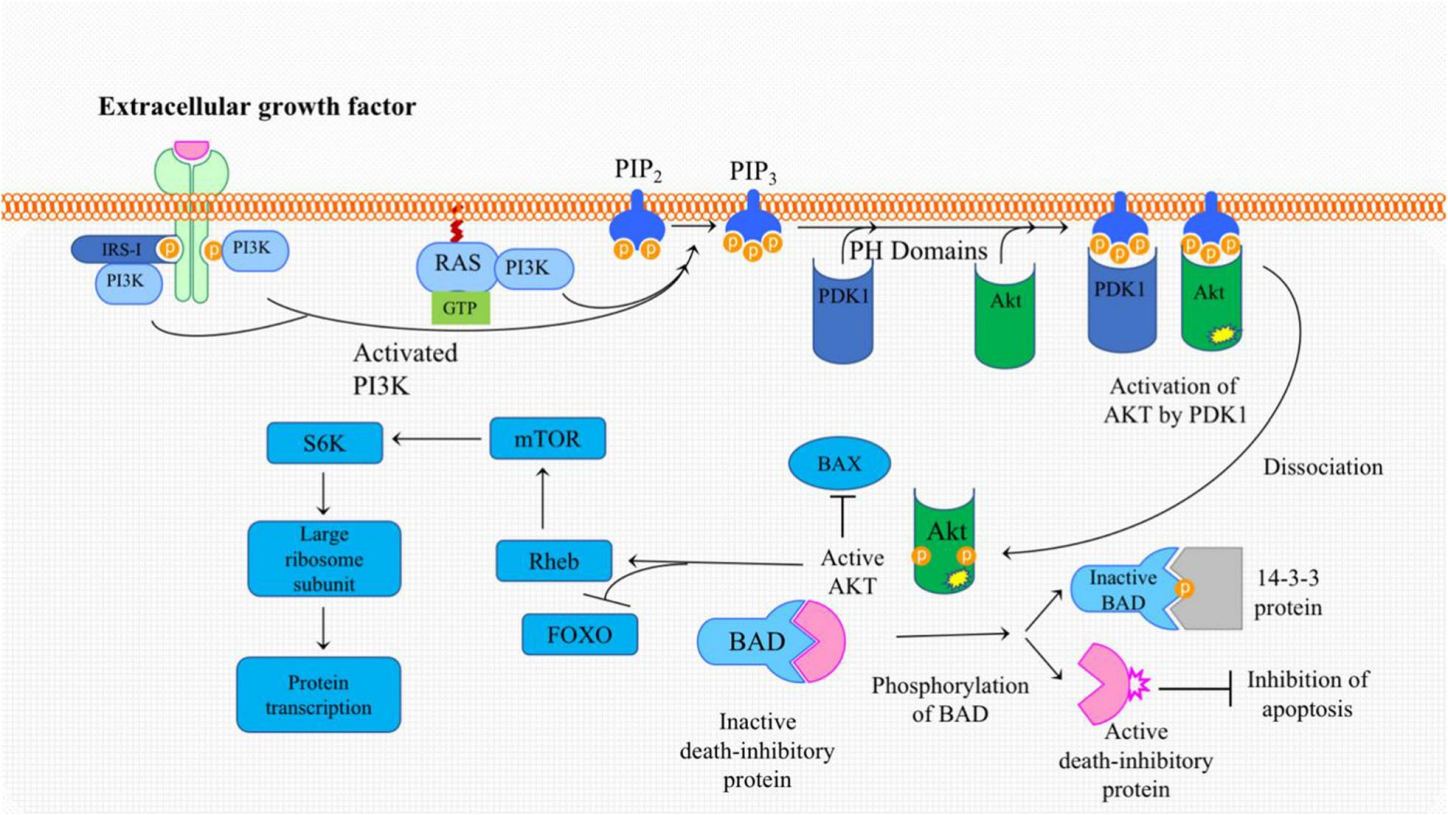
Activation of PI3K: Binding of an external ligand leads to the dimerization of receptor monomers and the heterologous autophosphorylation. Depending on the receptor, different proteins may bind to a phosphorylated domain. The insulin receptor substrate-1(IRS-1) binds to the activated IGF-1 receptor. IRS-1 serves as a binding and activation site for PI3K. In addition, PI3K may bind directly to a phosphorylated receptor tyrosine kinase, a completely different mechanism of PI3K. Activation begins with the small membrane bound GTPase Ras. By binding to active GTP-bound Ras, PI3K is activated, and migrates to the inner side of the cell membrane where it binds to phosphatidylinositol bisphosphate or PIP2. PI3K can phosphorylate PIP2 to PIP3, which can activate protein kinase B, also known as Akt. Akt binds to BAX and hinders its ability to form pores in the outer mitochondrial membrane, thus suppressing apoptosis. Moreover, Akt phosphorylates BAD leading to the release and activation of death inhibitory protein, which, as the name implies, inhibits apoptosis. Akt can also promote protein synthesis by first activating Rheb, which activates mTOR. mTOR itself interacts with and activates the translation factor S6K, thereby promoting mRNA translation and protein synthesis. In addition, Akt-induced phosphorylation of FOXO promotes the transfer of ubiquitin peptides onto the protein causing FOXO to undergoe proteasomal degradation

The second major downstream pathway of Ras is PI3K/Akt [34, 47]. PI3K is a lipid kinase that consists of a regulatory subunit, p85, and a catalytic subunit, p110. Ras interacts with p110 and recruits it to the cell membrane resulting in the activation of PI3-K, which in turn recruits phosphoinositide-dependent kinase-1 (PDK1). PDK1 or the mammalian target of rapamycin mTOR can then activate Akt [48]. Consequently, phosphorylated Akt drives several pathways that promote cellular growth and evasion of apoptosis [40] (Fig. 1).

Extensive studies have established the effects of statins on cell cycle progression, apoptosis, and senescence in different types of cells especially cancer cells and endothelial cells [49]. Interestingly, the cell-context appears to be a critical factor in determining the effect of statins on cell behavior. For instance, in endothelial cells, statins inhibit cytostatic and pro-apoptotic effects (see Table 1), whereas in cancer cells, the opposite effects are reported (Table 2). While the type of cells is an important parameter to consider in these discordant results, the concentrations/doses used could be the factor that tips the balance. Indeed, there is a large discrepancy among statin concentrations used in various studies. Pleiotropic effects of statins have been reported to appear at concentrations of 1–50 μM. However, at therapeutic doses, the mean concentration of statins in human serum only ranges from 1 to 15 nM. Additionally, 95–99% of statins in the blood are bound to proteins, therefore only 0.01–0.5 nM of them is the free fraction, and hence the pharmacologically active. Similarly, the therapeutic dosage in humans is approximately 0.1–1 mg/kg bodyweight, while doses of 1–100 or even 500 mg/kg body weight have been used in most studies in rodents. Indeed, statins concentrations used to induce pleiotropic effects in animal studies are much greater than those used in patients, reaching 1000 fold higher in some studies [50]. In this review, we discuss the effects of statins on endothelial cells (as models of non-cancerous cells) as well as on a battery of cancer cells.

## Cytoprotective effects of statins

In cells of cardiovascular origins such as endothelial cells, statins appear to protect against oxidative damages and apoptosis through a series of mechanisms.

## Cytoprotective effects of statins via reduction of free radicals’ production

The antioxidative activities of statins have been primarily ascribed to downregulation of ROS-generating enzymes such as NADPH oxidase and upregulating HO-1 rather than superoxide scavenging [51].

### Halting NADPH oxidase activity

ROS play critical roles in modulating various cellular processes and phenotypes [52, 53]. NADPH oxidase is considered the primary source of ROS, particularly superoxide radicals, in a multitude of cells [54]. The functional structure of NADPH oxidase consists of two membrane-bound components: gp91phox (Nox2) and p22phox, in addition to other cytosolic regulatory subunits including p40phox, p47phox, p67phox, and Rac. Phosphorylation and subsequent membrane translocation of the cytosolic subunits followed by interaction with p22phox and Nox2 are crucial steps in the activation of NADPH oxidase [55, 56]. The small GTP-binding protein Rac-1, a member of the Rho protein subfamily, plays a pivotal role in the assemblage and activation of the NADPH oxidase [57, 58]. In various cells including macrophages, human and rat smooth muscle cells, human vascular endothelial cells, cardiovascular cells, neuronal cells, cancer cells, and THP-1 derived monocytes statins have been shown by blocking prenylation of Ras and Rho families prevent the formation of NADP-oxidase subunits into a functional unit [59–67]. This reduces the production of ROS, and hence, a cytoprotective effect is achieved.

### Induction of HO-1 activity

The role of HO-1 as a cardio-vasculoprtective player has been established. It appears that by virtue of its ability to induce anti-inflammatory, anti-proliferative, anti-apoptotic, and antioxidative activities in the vasculature, HO-1 protects vessels from a multitude of pathologic conditions, prime of which is atherosclerosis [61]. HO-1 acts by catalyzing the oxidative degradation of heme to carbon monoxide, biliverdin, and free iron [68]. Importantly, statins have been shown to induce HO-1 activity, apparently via p38- and PI3K/Akt-dependent mechanism, as inhibition of these two pathways seem to abrogate statin-induced HO-1 expression. [59–61]. This is in line with other reports showing that statins act via PI3K/Akt to stabilize HO-1 mRNA [62, 65].

## Cytoprotective and cytotoxic effects of statins via activation PI3K/Akt in endothelial cells

**Fig. 2:**
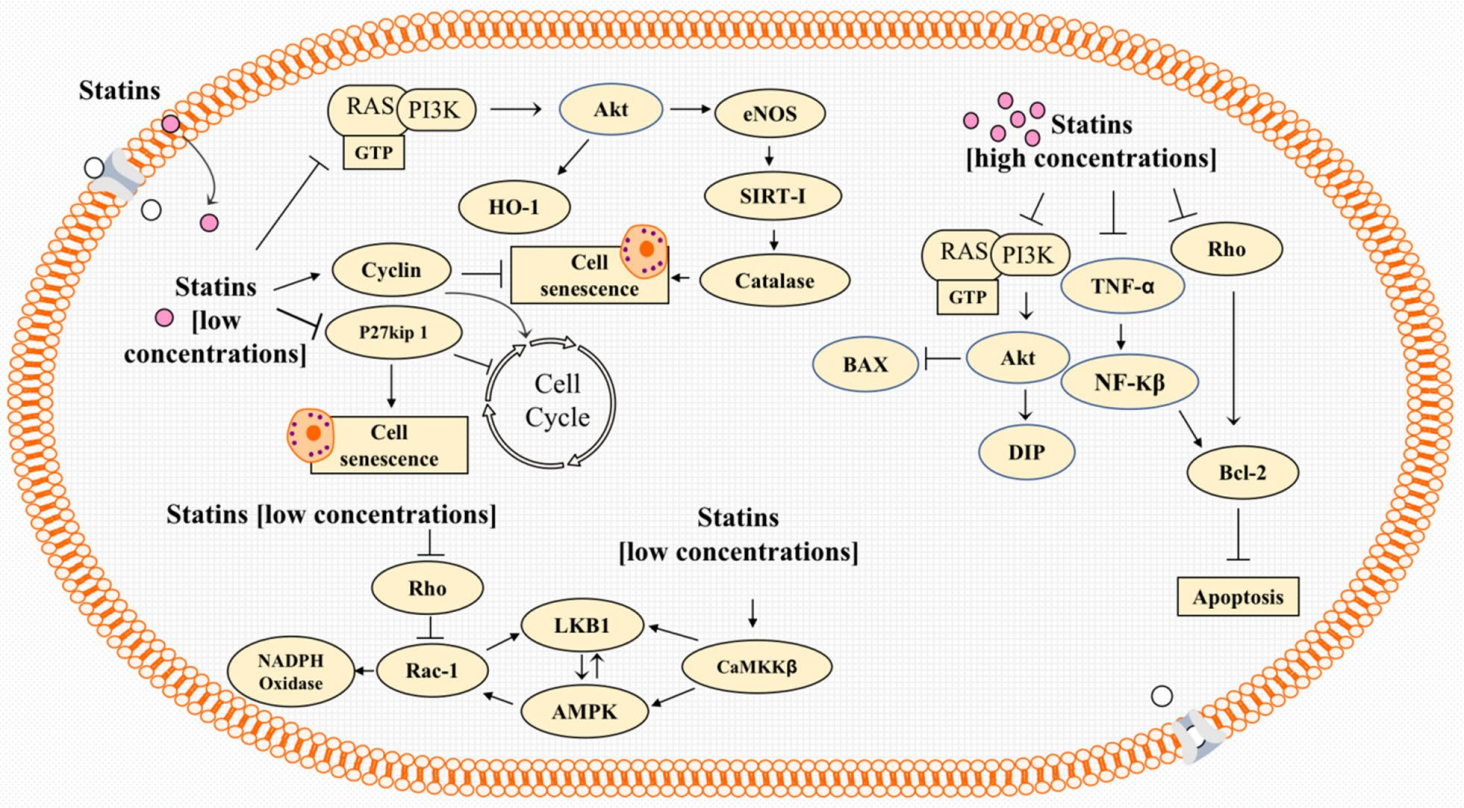
Low doses of statins (left-top) potentiate HO-1 activity via PI3K/Akt. In addition, PI3K/Akt stimulates eNOS, thereby causing increased catalase activity which prevents free radical-induced senescence. Moreover, eNOS upregulates VEGF, which is a main angiogenic factor. Low doses of statins induce cell cycle progression and reduce senescence by regulating the expression of several proteins including p27Kip1. They also activate caMKKβ which drives the activation of AMPK and LKB1. In addition, by virtue of its ability to stimulate Rac 1, AMPK can also regulate NAPDH oxidase and eNOS-mediated angiogenesis. An alternative pathway by which low doses of statins induce Rac-1 is by inhibiting Rho. On the other hand, high doses (top-right) induce apoptosis by inhibiting PI3K/Akt-modulated activity of death inhibitory protein (DIP) and BAX. Moreover, statins at high concentrations, can also induce apoptosis by inhibiting Rho-induced Bcl-2 or TNF-α-induced NFkB

PI-3 K/Akt and AMPK are two extensively studied pathways underpinning the anti-apoptotic effects of statins in noncancerous cells such as EPCs and HUVEC. These cytoprotective effects of statins have been studied particularly in endothelial cells, largely because of the ability of these drugs to modulate angiogenesis. In regard to concentrations used, it is important to cautions that statins act as a double-edged sword in endothelial cells and angiogenesis [69]. While nanomolar concentrations of statins induce angiogenesis in human umbilical vein endothelial cells, micromolar concentrations exert reverse effects [70], which has been attributed to the ability of these drugs to inhibit proliferation and migration as well as inducing apoptosis in these cells [71, 72]. Importantly, Akt appears to mediate these effects via a endothelial nitric oxide synthase (eNOS), a key regulator of vascular homeostasis [73–76]. Ineed, activated Akt stimulates post-transcriptional phosphorylation, hence activation, of eNOS which in turn activates the VEGF-mediated migration of mature endothelial cells and subsequently stimulates angiogenesis [69, 70, 77–79] (see Fig. 2).

Clinically relevant doses (0.01 to 0.1 µM) of atorvastatin activate endothelial Ras and promote Akt and eNOS phosphorylation activation. In contrast, higher concentrations (> 0.1 µM) of atorvastatin block angiogenesis and migration of endothelial cells by inducing apoptosis [69, 80].

*In vitro*, atorvastatin or mevastatin (0.1, 0.05, and 0.01 µM) inhibits the onset of endothelial progenitor cell senescence in a dose-dependent manner. Moreover, atorvastatin increases EPC proliferation and colony-forming capacity. GGPP, mevalonate, and FPP reverse the senescence inhibitory effect of atorvastatin. Contextually, atorvastatin’s anti-senescence effect appears to be to an increase in the expression of cell cycle-promoting genes including cyclins as well as suppression of p27Kip1, a cell cycle-inhibitory protein [81] (Figure 2). Likewise, pitavastatin induces migration, proliferation, and viability of human microvascular endothelial cells (HMVECs) at low concentrations (0.01 mM) but suppresses these cellular processes at higher concentrations (1 mM) [59, 82]. At nanomolar concentrations, atorvastatin, pravastatin, and pitavastatin suppressed hydrogen peroxide-induced senescence in human umbilical vein endothelial cells (HUVECs). This effect occurred via statin’s ability to activate Akt and subsequently upregule eNOS, SIRT1, and catalase. [83] (Figure 2).

**Fig. 3:**
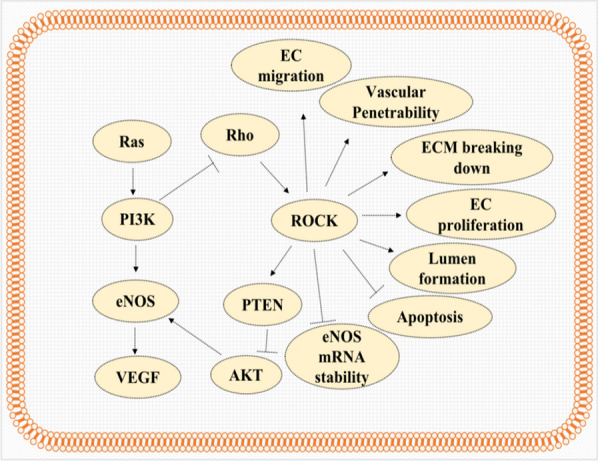
Role of Rho in angiogenesis in Endothelial cells. The activation of the Rho/ROCK pathway via induction of phosphatase activity of PTEN, which in turn inhibits the activation of Akt and via reducing the stability of eNOS mRNA abolishes the acute activation of angiogenesis by statins; however, this pathway induces all process involved in angiogenesis including endothelial (ES) migration, vascular permeability, extracellular matrix (ECM) degradation, endothelial cell (EC) proliferation, lumen formation, and inhibition of apoptosis; therefore in long term period, Rho/Rock pathway may increase angiogenesis by statins

Despite the aforementioned evidence, high concentrations of statins can evoke endothelial release of VEGF and an increase in endothelial apoptosis, most probably by inhibition of geranylgeranylation of Rho, which is known to play a paradoxical role in angiogenesis. Indeed, Rho modulates the activity of VEGFR-2, which is employed by VEGF to activate Rho GTPases [71, 72, 84]. Rho GTPases can then act via regulatory and effector proteins, most notable of which is ROCK, to influence angiogenic processes [85, 86] (Fig. 3).

Interestingly, statins upregulate the expression of eNOS via inhibition of Rho activity [77, 87–89]. Despite the angiogenic functions, Rho GTPase may downregulate eNOS expression via destabilizing its mRNA. As such, statins upregulate the expression of eNOS by prolonging its mRNA half-life through inhibiting the Rho/ROCK pathway [86]. Contextually, inhibition of the Rho/ROCK pathway activates PI3K/Akt and leads to the rapid phosphorylation (acute) activation of eNOS [86, 90]. This suppression of PI3K/Akt is assumed to arise from decreasing PTEN activity, since RhoA/ROCK is required for intracellular localization and phosphorylation of PTEN, which in turn is crucial for the phosphatase activity of PTEN that antagonizes the PI3K/Akt pathway [91]. Therefore, despite its pro-angiogenic role, the Rho/ROCK pathway may negatively regulate the acute activation of angiogenesis via two distinct mechanisms, namely, eNOS expression and eNOS activity [86].

## Cytoprotective effects of statins through activating AMPK in endothelial cells

AMPK (AMP-activated protein kinase) is a cellular energy sensor that is activated in response to an increase in the intracellular AMP: ATP ratio. It stimulates ATP- producing catabolic pathways and inhibits ATP- consuming anabolic pathways. For instance, AMPK inhibits fatty acid and cholesterol synthesis through direct phosphorylation of anabolic enzymes including Acetyl-CoA carboxylase (ACC) and HMG-CoA reductase (HMGR) [92]. While the AMPK pathway is traditionally thought of as a regulator of metabolism, recent studies have demonstrated that AMPK may play a significant part in maintaining normal endothelial function through AMP-independent activation of AMPK [75, 93–96]. LKB1 and the calcium/calmodulin-dependent kinase (CaMKK) are protein kinases that phosphorylate AMPK. The pathways that regulate LKB1 remain elusive. CaMKK seems to play a key role in modulating effects of statins by phosphorylation of LKB1 and AMPK, as well as by direct activation of Rac-1 [97](see Fig. 2).

In cultured vascular endothelial cells (e.g. ovine aortic endothelial cells), simvastatin (10 μM) increased activity of Rac-1 via CaMKKβ, AMPK and LKB1. The importance of Rac-1 aictivation becomes evidence in light of the notion that Rac-1 plays a key role in eNOS activation. Indeed, siRNA-mediated AMPK knockdown was shown to suppress Rac-1 activation and subsequently prevents activation of eNOS. Interestingly, it has been shown that Rac-1 in turn regulates LKB1 phosphorylation [98].

Activation of small GTPases like Rho and Rac-1 requires geranylgeranylation and subsequent translocation to the cell membrane. Interestingly, some studies showed that statins, by inhibiting, prenylation, paradoxically activate Rac-1 [99]. For example, simvastatin (10 μM) caused a 34-fold increase in Rac-1 activation in endothelial cells despite its inhibition of prenylation and activity of Rho [98]. One plausible explanation for this paradoxical observation is that statins, by reducing geranylgeranylation, preferentially inhibit Rho, which is a tonic inhibitor of Rac-1. This inhibition effectively leads to Rac-1 activation [100]. Alternatively, it is possible that statins dissociate the inhibitory interaction of Rac-1 with guanine nucleotide dissociation inhibitors (RhoGDI) [98].

## Cytotoxic (apoptotic) and cytostatic effects of statins in cancer cells

Statins can induce apoptosis via different mechanisms [39]. Indeed, they can activate the intrinsic pathway of apoptosis via disturbing the mitochondrial membrane potential and releasing the second mitochondria-derived activator of caspases (Smac/DIABLO) [116]. Moreover, upregulation of proapoptotic proteins Bax and Bim besides the downregulation of antiapoptotic protein Bcl-2 are considered the main mechanisms of induction of apoptosis by statins. Statins have also been revealed to activate procaspase 3, 7, 8, and 9 [78, 116–118].

The effects of statins’ concentrations on Bcl-2 have been studied widely. Indeed, in high concentrations, statins may induce apoptosis by reducing Bcl-2 level, while in lower concentrations, they tend to suppress apoptosis and cell death by increasing Bcl-2 expression [119]. (Fig. 2). As is known, the expression of Bcl-2 gene has been shown to be upregulated by NF-κB. In this context, it is noteworthy that high concentrations of simvastatin (50 μM) were found to reduce Bcl-2 protein levels through inhibition of TNF-α, protein that is for NF-κB activation [120]. Similarly, simvastatin (20 μM) was shown to reduce Bcl-2 mRNA and induce apoptosis in a battery of human cancer cell lines including MCF7 breast cancer cells, NCI-N87 human gastric cancer cells, HepG2 human hepatocellular carcinoma and non-small cell lung carcinoma (NCH lung) cells. However, normal cells (SAEC human normal small airway epithelial cells) did not seem to exhibit the same response [121]. Other lipophilic statins (1 μM for cerivastatin and 10 μM for atorvastatin and simvastatin) promoted apoptotic programs by inhibiting RhoA activity, which caused decreased phospho-p42/p44-MAPK and Bcl-2 levels [122].

Cytostatic effects of statins on cancer cells have also been reported. These effects occur mainly via the upregulation of cell cycle inhibitors including p21*WAF1/CIP1* or p27KIP1 [42]. Simvastatin (10 μM) downregulates the transcriptional activity of ATF-2 and c-jun, which then causes a dramatic decrease in the proliferative capacity of glioma cells [123].

There are many studies indicating the major mechanism underlying the cytotoxic and cytostatic effects of statins on cancer cells arise from reduction of geranylgeranyl pyrophosphate (GGPP) which is crucial for membrane localization and activation of small G proteins like Rho. These studies report that supplying cells with mevalonate or GGPP reverses the inhibitory effect of statins and prevent induction of apoptosis or cell cycle arrest by statins. Lovastatin (0.3 µM) was shown to induce senescence and G1 cell cycle arrest in human prostate cancer cells, and supplementation with GGPP or mevalonate, but not FPP, reversed cell cycle arrest and senescence. In addition, constitutively active RhoA (caRhoA) reversed lovastatin-induced senescence in caRhoA-transfected PC-3 cells. This indicates that statins could act through the inhibition of Rho activity to induce cytotoxic effects, at least in this cell line [124].

Pitavastatin (10 μM) enhanced the effects of radiation on cellular senescence in breast cancer and melanoma tumors. However, 5 mM mevalonic acid was sufficient to restore these effects of pravastatin [30]. Likewise, simvastatin (100 nM) was shown to exert cytostatic and senescent effects and partially induce apoptosis in prostate epithelial cells. In contrast, 10 μM simvastatin had a cytotoxic effect both on normal and cancer cells. A combination of LDL-cholesterol and mevalonate supplementation was able to rescue the cytostatic/cytotoxic of 10 μM simvastatin [125]. Others report that lovastatin (0.3 µM) causes senescence and G1 cell cycle arrest in human prostate cancer cells. GGPP or mevalonate, but not FPP, reversed the cell cycle arrest and cell senescence induced by lovastatin. Moreover, constitutively active RhoA (caRhoA) abolishes the senescence induced by statins in caRhoA-transfected PC-3 cells [124]. Table 2 provides highlights and an overview of further studies regarding the effect of statins on cancer cells.

## Conclusion and perspective

The effects of statins on endothelial cells can be either protective via boosting cytoprotective effects such as antioxidant levels in the cells, or destructive via inhibition of growth signaling and induction of apoptosis. Although the type of effect depends on the applied concentration, an absolute threshold/dose could not be definitively determined. However, it seems that at lower doses -particularly in nanomolar concentrations—statins act more in line with cytoprotection rather than halting the cell cycle or inducting apoptosis in ECs. Moreover, it seems that the effects of different concentrations of statins on endothelial cells largely depends on their effects on the Rho activity. Negative feedback mechanisms may be yet another underlying mechanism that could explain the contradictory effects of statins in cancer cells versus EPCs. Statins block the production of isoprenoid units required for the prenylation of proteins, which ultimately results in the deceleration of cell cycle progression. The reduced rate of cell cycle progression is sensed by the cell, which then reduces the expression and activity of cell cycle inhibitory proteins as a feedback response. Afterward, the cell cycle is regulated by the positive inducers (proto-oncogenes) like the Ras superfamily rather than negative regulators (tumor-suppressors) such as p53 and p27.

Cancer cells normally have a high proliferation rate and, as a result, there may be permanently increased levels of cytostatic proteins; however, cancer cells are not responding to high levels of these proteins. Therefore, low concentrations of statins probably are not able to exert the same effects in EPCs and cancer cells. However, how low concentrations of statins can induce apoptosis in cancer cells is a question yet to be answered. As a possible mechanism, cancer cells may be more dependent on cell cycle stimulatory factors and even the low concentration of statins can impose cytostatic effects. To determine the precise effects of statins on cancer cells, the effects of the nanomolar concentrations of these medications on these cells should be further studied.

## Data Availability

No primary data exists for this review article.
